# A Rare Case of Unilateral Xanthogranulomatous Ureteritis Mimicking an Inferior Vena Cava Tumor

**DOI:** 10.7759/cureus.34388

**Published:** 2023-01-30

**Authors:** Varsha R Bhatt, Pranav G Jawade, Vikas Jadhav, Amit Palange, Anusha Vattikuti

**Affiliations:** 1 General Medicine, Dr. D. Y. Patil Medical College, Hospital & Research Centre, Pune, IND; 2 Radiodiagnosis, Dr. D. Y. Patil Medical College, Hospital & Research Centre, Pune, IND

**Keywords:** xanthogranulomatous pyelonephritis, ureteritis, usg guided, ivc tumor, xanthogranulomatous

## Abstract

Xanthogranulomatous pyelonephritis (XPG) is a known clinical entity; however, the further progression of this inflammatory pathology to adjacent organs, including the ureter, bladder and urethra, is extremely rare. Xanthogranulomatous inflammation of the ureter is a chronic inflammatory state where foamy macrophages are seen in the lamina propria along with multinucleated giant cells and lymphocytes forming a granulomatous inflammation, which is benign. Based on its appearance on computed tomography (CT) scan images, it can easily be misidentified as a malignant mass, and the patient can be subjected to surgery that can lead to complications. Here we present a case of an elderly male with a known case of chronic kidney disease with uncontrolled type 2 diabetes mellitus who presented with fever and dysuria. Upon further radiological investigations, the patient had underlying sepsis and was seen to have a mass involving the right ureter and inferior vena cava. Upon biopsy and histopathology, he was diagnosed with xanthogranulomatous ureteritis (XGU). The patient underwent further treatment and was followed up.

## Introduction

Xanthogranulomatous ureteritis is a rare clinical entity [1]. Its renal counterpart, xanthogranulomatous pyelonephritis, is a relatively known pathology, a granulomatous inflammation, which damages the renal parenchyma with infiltration of lipid-laden macrophages, plasma cells, and multinucleated giant cells in the renal parenchyma on histopathology [2]. Patients with recurrent urinary tract infections, urinary tract and renal obstruction, and Gram-negative urosepsis are susceptible to xanthogranulomatous inflammation. Based on its appearance in imaging modalities, it can often be misdiagnosed as a malignant mass; hence, a better understanding of this condition can lead to better outcomes. We present this case of an elderly male who was a known case of uncontrolled diabetes mellitus and presented with dysuria. This extremely rare concurrent ureteral granulomatous inflammation involving the inferior vena cava was found in subsequent investigations.

## Case presentation

A 65-year-old male came to medicine OPD with complaints of increased frequency of micturition for 15 days and a history of mild fever associated with acute chills. The patient was experiencing generalized weakness and loss of appetite for seven days. The patient was a known case of type 2 diabetes mellitus with diabetic nephropathy, which is chronic kidney disease, and had no history of hemodialysis. He was hospitalized 10 days back for uncontrolled diabetes and was discharged. A year ago, the patient was hospitalized for diabetic ketoacidosis and COVID-19 pneumonia. The patient was a chronic alcoholic and tobacco chewer for almost 40 years. The patient was admitted to the hospital and underwent a series of radiological and blood investigations. Blood and urine cultures were sent after admission. On admission, his blood pressure was 160/90 mmHg and his heart rate was 96 beats per minute. 

The patient’s hemoglobin was 9.4 g/dl, platelet count was 3,40,000/μl, and total leucocyte count was 21,400/μl. His creatinine was 6.31 mg/dl and urea was 158 mg/dl. The patient’s liver function test was within normal limits. His c-reactive protein was 84.10 ng/l, serum procalcitonin was 2.06 mcg/ml, and erythrocyte sedimentation rate was 90 mm/h. His glycated hemoglobin A1c (HbA1c) was 9.2%. The patient’s blood culture was sent, and *Klebsiella pneumoniae* was isolated, which was sensitive only to fosfomycin and tigecycline (Table 1).

**Table 1:** Laboratory investigations of the patient HbA1c: glycated hemoglobin A1c

Test	Result	Reference range
Hemoglobin	9.4 g/dl	13.2-16.6 mg/dl
Total leucocyte count	21,400/µl	4,000-10,000 µl
Serum creatinine	6.31 mg/dl	0.6-1.2 mg/dl
Serum urea	158 mg/dl	17-49 mg/dl
C-reactive protein	84.10 mg/l	<2 mg/l (low cardiovascular risk)
Serum procalcitonin	2.06 ng/ml	<0.08 ng/ml (for males)
Erythrocyte sedimentation rate	90 mm/h	Upto 20 mm/h (for males of age 50-85 years)
Blood culture	Klebsiella pneumoniae	-
Urine culture	Polymicrobial growth	-
Fasting blood glucose	186 mg/dl	<100 mg/dl (normal 100-125 mg/dl for prediabetes)
Postprandial blood glucose	321 mg/dl	<140 mg/dl (normal 140-199 mg/dl for prediabetes)
HbA1c	9.2%	4.0-5.6%

On urine routine microscopy, the patient had 50-60 pus cells, and urine culture and sensitivity was suggestive of polymicrobial growth. The patient’s inflammatory markers were followed up in days to come. The patient’s daily urine output was monitored and charting was done on an hourly basis. The patient was not dialyzed as his urine output was adequate. Renal function test was also done on daily basis and there was no further increase in serum urea and creatinine levels.

**Figure 1:**
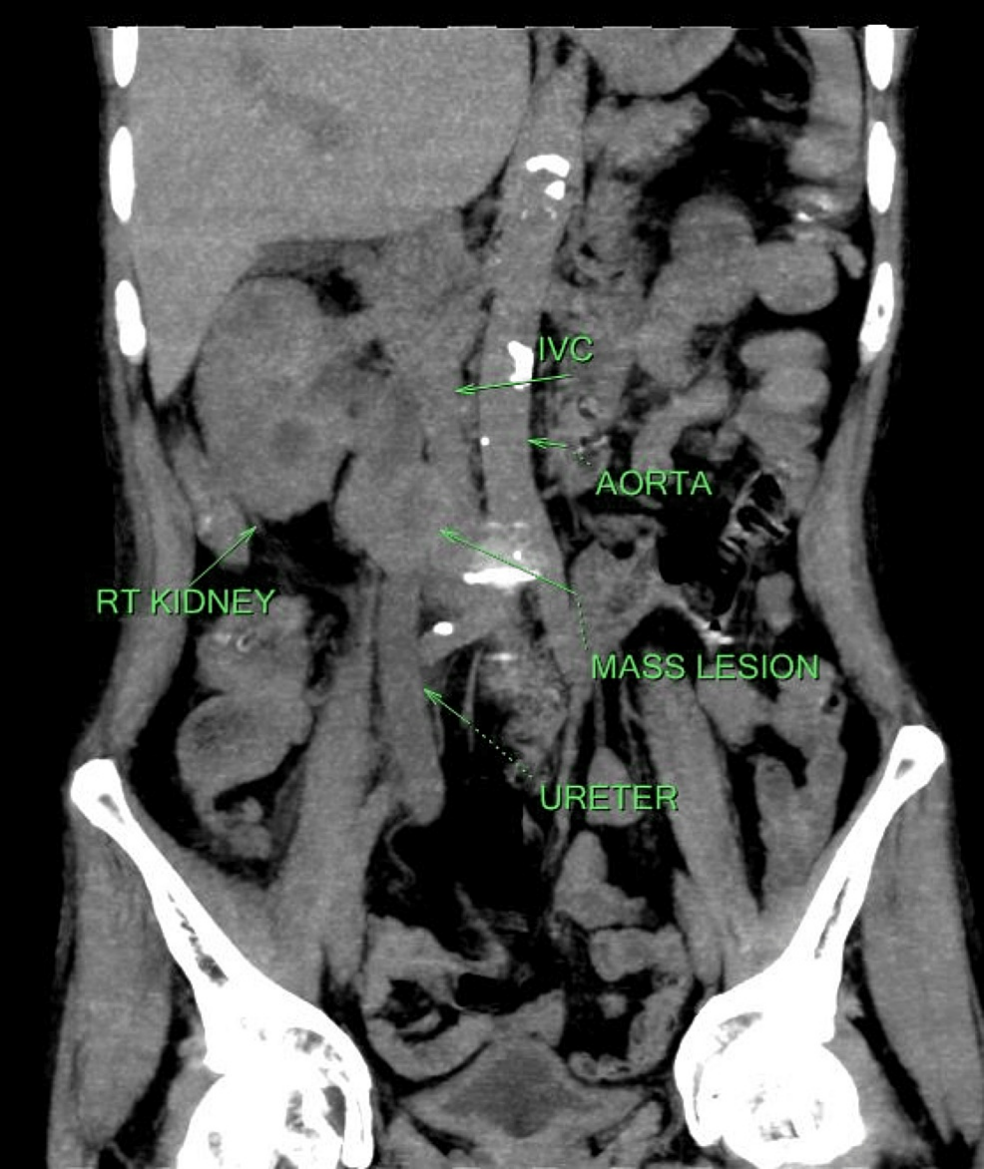
Non-contrast computed tomography scan showing mass lesion along with right ureter and inferior vena cava

Ultrasound sonography of the abdomen and pelvis suggested hydronephrosis with raised cortical echogenicity and altered corticomedullary differentiation. Mild perinephric soft tissue edema was also seen, along with mild, turbid fluid collection in the right renal pelvi-calyseal system suggesting pyonephrosis. The right upper one-third hydro-ureter was also visualized with a mildly thickened and edematous wall and showed a nearly obstructing oval heterogeneous upper one-third ureteric intramural encasing mass lesion of size approximately 24 x 23 x 20 mm that showed absent peristalsis and was indenting and infiltrating the adjacent inferior vena cava. The left kidney showed altered corticomedullary differentiation, and the left ureter was regular in its course. A non-contrast computed tomography (NCCT) scan was done (Figure 1).

**Figure 2:**
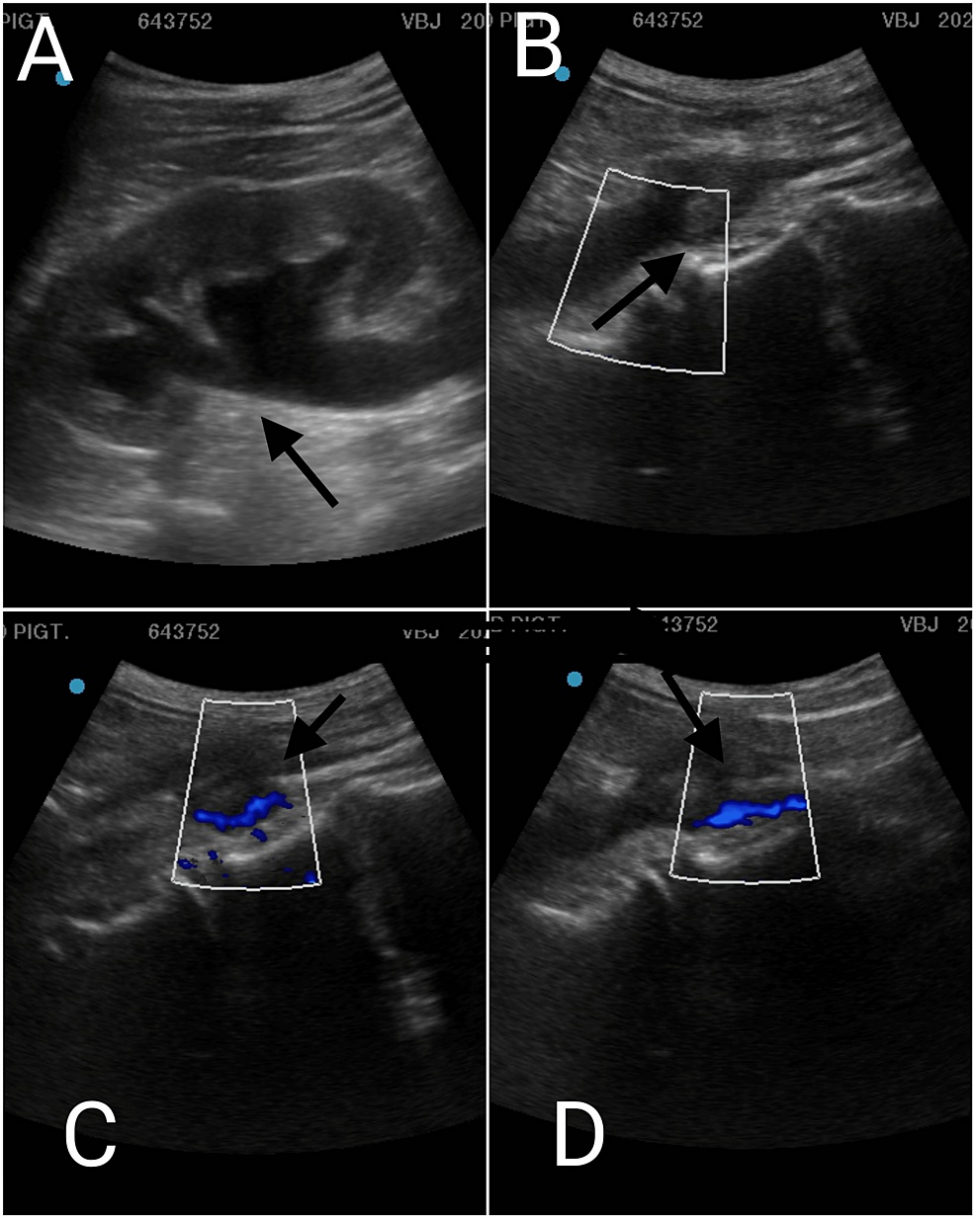
Doppler ultrasound of the patient (A) Ultrasound of abdomen showing hydronephrosis. (B) Color Doppler image showing ureter along with mass lesion. (C) Color Doppler image showing the upper one-third of ureter and mass with inferior vena cava. (D) Color Doppler image showing the mass compressing and infiltrating the lumen of the inferior vena cava.

It was suggestive of a well-defined solid soft tissue density mass lesion measuring approximately 31 x 37 x 32 mm, in the lumen of the right ureter at the L3 level with extra luminal extension. Fat planes between the lesion and adjoining inferior vena cava were obscured. Extension of soft tissue density lesion along the course of inferior vena cava lesion, possibly a neoplastic mass (leiomyosarcoma) arising from the inferior vena cava with adjoining ureteric involvement or vice versa with proximal obstructive uropathy was suggested by the radiologist. Another differential was the possibility of thick purulent material. On Doppler ultrasound, a ureteral mass was seen compressing and infiltrating the inferior vena cava when performing a biopsy (Figures 2A-2D).

**Figure 3:**
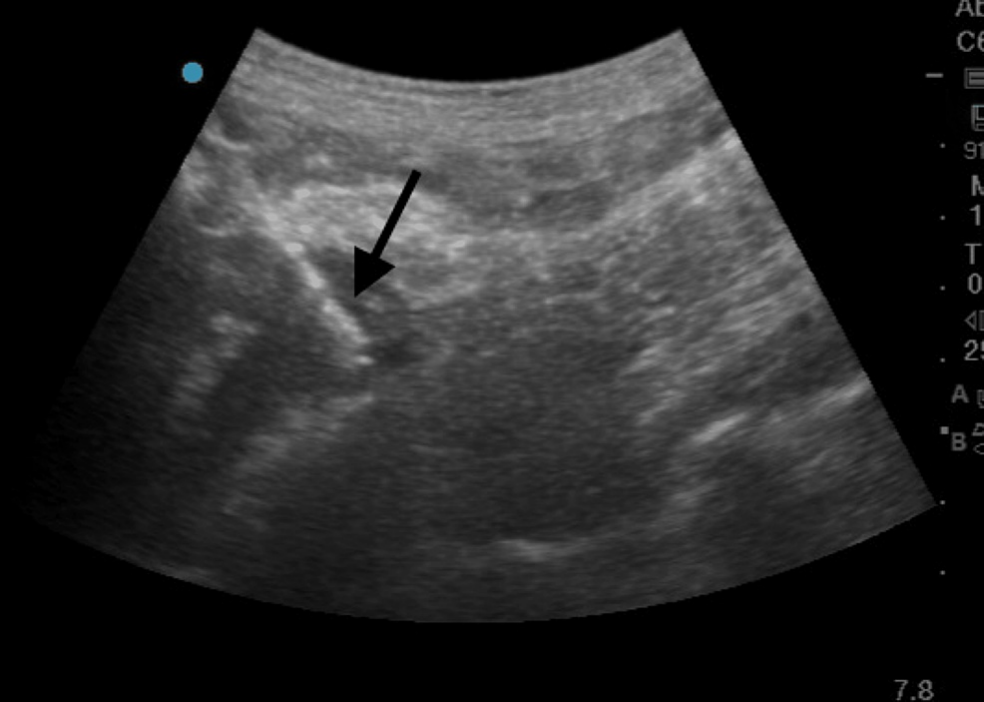
Ultrasonography-guided biopsy of mass lesion

Biopsy was taken with precision (Figure 3). The patient received intravenous meropenem and tigecycline as per the antibiotic susceptibility report for 14 days, along with 3 g of oral fosfomycin at days 0, 3, and 7. In the antibiotic susceptibility report the minimal inhibitory concentration (MIC) of meropenem was ≥1 mcg/ml. The MIC of tigecycline was 2 mcg/ml and MIC of fosfomycin was ≥64 mcg/ml. The *Klebsiella pneumoniae* strain isolated in the blood culture was found to be susceptible to these antibiotics. The patient was also on short-acting insulin per the sliding scale for diabetes mellitus and oral amlodipine 5 mg twice a day for hypertension. Throughout his stay in the hospital, his urine output was monitored; since he had an output of 700-1,000 ml approximately per day, he never underwent hemodialysis. Nephrology and urology consultation was done. The patient also underwent an ultrasonography-guided biopsy of a ureteric and inferior vena cava mass-like lesion, which was further sent for histopathology (Figure 3). It was suggestive of fibro collagenous tissue admixed with inflammatory cells comprised of lymphocytes, plasma cells, foamy histiocytes, and a few eosinophils.

**Figure 4:**
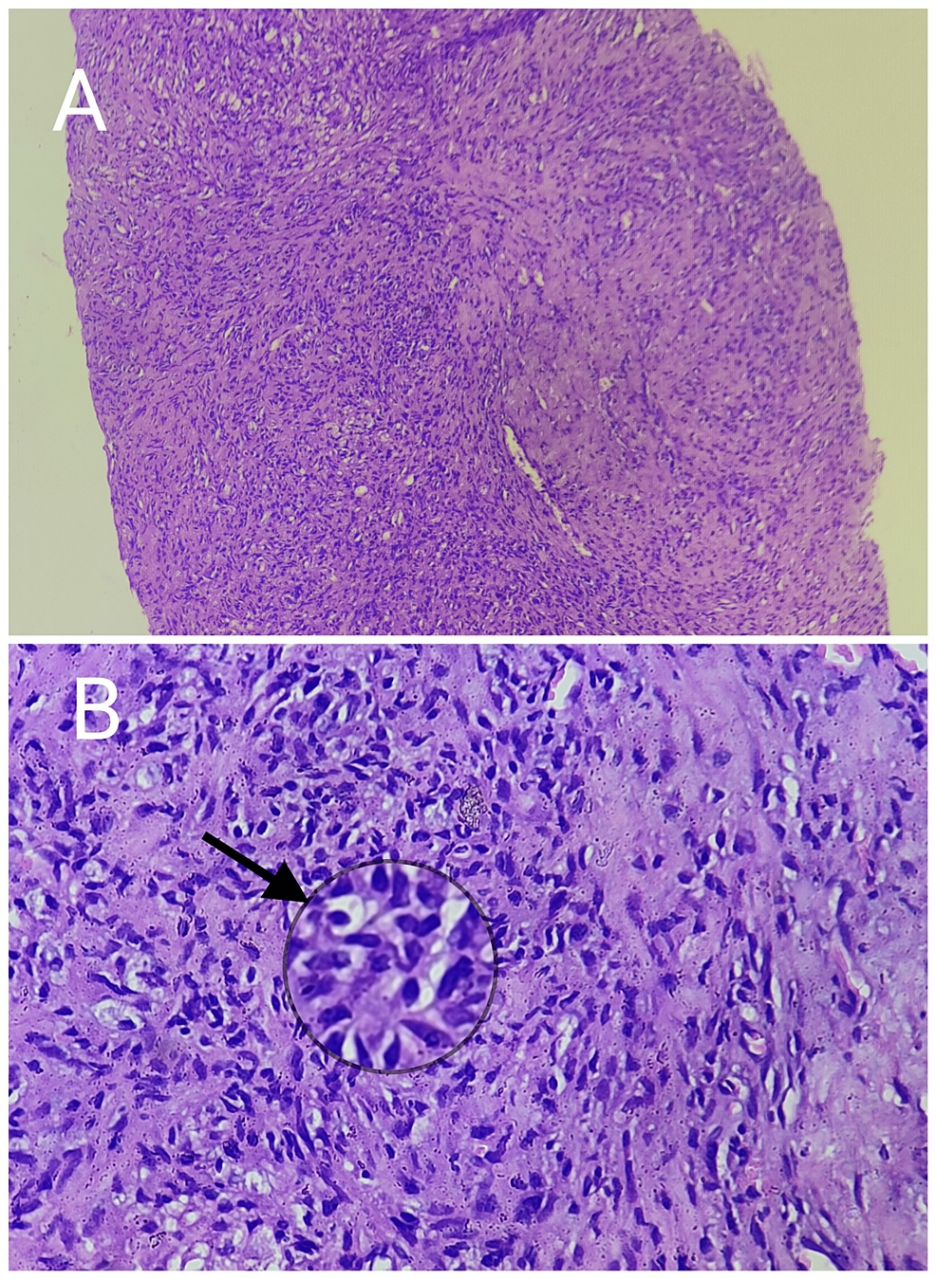
Low- and high-power scanner images (A) Low-power scanner image showing a core biopsy in which dense mononuclear inflammatory infiltrate can be appreciated (100x). (B) Higher magnification image showing a well-formed granuloma with numerous foamy histiocytes and macrophages representing a chronic inflammatory process (400x).

**Figure 5:**
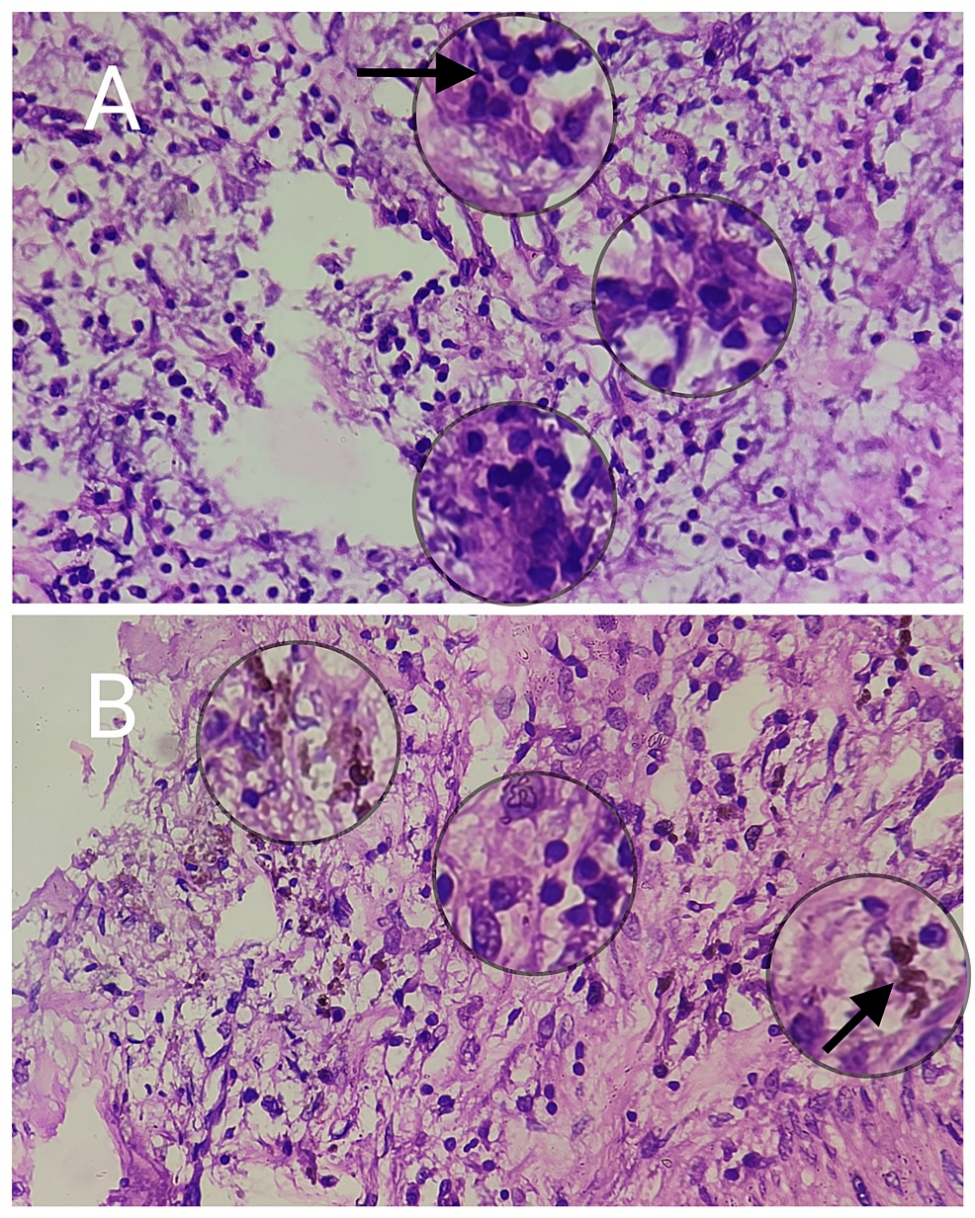
Higher magnification (400x) images showing scattered characteristic multinucleated Touton giant cells (A) and focal deposition of hemosiderin pigment (B).

Focally, the foamy histiocytes were seen forming clusters with Touton giant cells. The intervening stroma showed focal proliferation of spindle cells with centrally placed bland nuclei resembling a myofibroblast. Focal hemosiderin pigmentation was noted as well. No evidence of increased or atypical mitosis, caseous necrosis, epitheloid granuloma, or malignancy was seen in the section and thus was suggestive of a xanthogranulomatous inflammation (Figures 4A, 4B and Figures 5A, 5B).

Ultrasonography-guided right kidney percutaneous nephrolithotomy (PCNL) with pigtail insertion and double-J (DJ) stent insertion were done. The patient was on oral antibiotics, a single dose of 3 g of fosfomycin and tablet linezolid 600 mg orally for a week, and strict glycemic and blood pressure control was advised and monitored.

Finally, the patient was discharged after two successive repeat negative blood and urine cultures and was advised to follow-up after one week. At the time of discharge, his c-reactive protein was 11.20 ng/l and serum procalcitonin was 0.21 mcg/ml. He was followed up in weeks to come, and the indwelling pigtail in the right kidney was removed after three weeks. Considering his chronic kidney disease status, the patient was not subjected to a nephroureterectomy and underwent conservative medical management further. Informed consent from the patient was taken.

## Discussion

Xanthogranulomatous inflammation is a commonly seen pathology in the pancreas, appendix, gallbladder, ovary, endometrium, and kidneys [3]. Xanthogranulomatous ureteritis is a rare extension of its counterpart, xanthogranulomatous pyelonephritis, a well-encountered pseudotumor-like mass-forming inflammatory entity in clinical settings [2,4]. Xanthogranulomatous pyelonephritis has been classified into three stages, namely, nephric (stage I), nephric and perinephric (stage II), and nephric, perinephric and paranephric (stage III) [5]. It is commonly associated with urinary tract infections mainly caused due to Gram-negative bacteria like *Escherichia coli*, *Proteus mirabilis*, or less commonly, *Staphylococcus aureus*, Group B Streptococcus, Candida, Klebsiella, or Bacteroides and also anaerobic infections [6,7]. It is more commonly seen in middle-aged females than in males [2,8]. Recurrent urinary tract obstruction is a common predisposing factor, as seen in the oldest case reports like the one published by Remigio et al. [9]. As mentioned in a case report by Rodgers and Williamson, its association with *Klebsiella pneumoniae* is extremely rare and similar to our case [2]. Computed tomography (CT) is the imaging modality of choice. It can help in the preoperative assessment of the inflammatory condition with its extent, and on CT, liposarcoma and angiomyolipoma or lipoma are the closest differentials [10]. Ultrasound can also help us diagnose this pathology [8]. Surgical resection is often considered a curative treatment for xanthogranulomatous inflammation [11]. However, in our case, we decided to perform a diagnostic biopsy which helped us diagnose it. Further management includes surgical resection of the granulomatous tissue, like partial ureterectomy. Failure to do so can lead to further spread and formation of cutaneous fistulae [12].

## Conclusions

Increasing awareness among physicians, urologists, radiologists, and pathologists about this entity is essential. Extensive use of radio imaging tools, histopathological analysis, and a biopsy can help diagnose this benign pathological entity. Surgical resection is the further line of management; however, rather than subjecting the patient to surgical resection or nephroureterectomy, a case-by-case approach has to be taken to prevent unwanted complications. Similar concurrent involvement of the bladder and urethra should also be carefully examined. Meticulous medical management of the underlying sepsis and thorough radiological investigations can help in early diagnosis and better management of the condition.

## References

[1] Mandal AK, Vaiphei K, Bhusnurmath SR, Vaidyanathan S (1989). Ureteral involvement in stage I xanthogranulomatous pyelonephritis - (a case report). J Postgrad Med.

[2] Rodgers SA, Williamson SR (2021). Xanthogranulomatous ureteritis mimicking ureteral involvement by cancer in a radical cystectomy specimen. Int J Surg Pathol.

[3] Pottakkat B, Saxena R, Nag HH, Kumari N, Krishnani N (2006). Ampullary xanthogranulomatous inflammation mimicking periampullary cancer: report of a case. JOP.

[4] Itano NB, Lightner DJ, Brandt KR, Sebo TS (2000). Xanthogranulomatous urethritis: a case report. J Pelvic Med Surg.

[5] Malek RS, Greene LF, DeWeerd JH, Farrow GM (1972). Xanthogranulomatous pyelonephritis. Br J Urol.

[6] Li L, Parwani AV (2011). Xanthogranulomatous pyelonephritis. Arch Pathol Lab Med.

[7] Fornari A, Dambros M, Telöken C, Hartmann AA, Kolling J, Seben R (2007). A case of xanthogranulomatous cystitis. Int Urogynecol J Pelvic Floor Dysfunct.

[8] Sharma S, Jhobta A, Goyal D, Sumala Sumala, Negi A (2006). Ureteral involvement in xanthogranulomatous pyelonephritis - rare manifestation. Indian J Radiol Imaging.

[9] Remigio P, Ramos C, Basirico P (1975). Xanthogranulomatous ureteritis. J Urol.

[10] Frederick MG, Hall BP (1995). Genitourinary case of the day. Replacement lipomatosis of the kidney. AJR Am J Roentgenol.

[11] Hayashi N, Wada T, Kiyota H, Ueda M, Oishi Y (2003). Xanthogranulomatous cystitis. Int J Urol.

[12] Mittal MK, Sureka B, Sinha M, Thukral BB (2013). Unilateral xanthogranulomatous pyelouretritis with a functioning kidney. Indian J Nephrol.

